# MirDIP 5.2: tissue context annotation and novel microRNA curation

**DOI:** 10.1093/nar/gkac1070

**Published:** 2022-12-01

**Authors:** Anne-Christin Hauschild, Chiara Pastrello, Gitta Kirana Anindya Ekaputeri, Dylan Bethune-Waddell, Mark Abovsky, Zuhaib Ahmed, Max Kotlyar, Richard Lu, Igor Jurisica

**Affiliations:** Department of Medical Informatics, University Medical Center Göttingen, Georg-August University, Göttingen, Lower Saxony 37075, Germany; Osteoarthritis Research Program, Division of Orthopedic Surgery, Schroeder Arthritis Institute and Data Science Discovery Centre for Chronic Diseases, Krembil Research Institute, University Health Network, Toronto M5T 0S8, Canada; Department of Medical Informatics, University Medical Center Göttingen, Georg-August University, Göttingen, Lower Saxony 37075, Germany; Osteoarthritis Research Program, Division of Orthopedic Surgery, Schroeder Arthritis Institute and Data Science Discovery Centre for Chronic Diseases, Krembil Research Institute, University Health Network, Toronto M5T 0S8, Canada; Osteoarthritis Research Program, Division of Orthopedic Surgery, Schroeder Arthritis Institute and Data Science Discovery Centre for Chronic Diseases, Krembil Research Institute, University Health Network, Toronto M5T 0S8, Canada; Osteoarthritis Research Program, Division of Orthopedic Surgery, Schroeder Arthritis Institute and Data Science Discovery Centre for Chronic Diseases, Krembil Research Institute, University Health Network, Toronto M5T 0S8, Canada; Osteoarthritis Research Program, Division of Orthopedic Surgery, Schroeder Arthritis Institute and Data Science Discovery Centre for Chronic Diseases, Krembil Research Institute, University Health Network, Toronto M5T 0S8, Canada; Osteoarthritis Research Program, Division of Orthopedic Surgery, Schroeder Arthritis Institute and Data Science Discovery Centre for Chronic Diseases, Krembil Research Institute, University Health Network, Toronto M5T 0S8, Canada; Osteoarthritis Research Program, Division of Orthopedic Surgery, Schroeder Arthritis Institute and Data Science Discovery Centre for Chronic Diseases, Krembil Research Institute, University Health Network, Toronto M5T 0S8, Canada; Departments of Medical Biophysics and Computer Science, and Faculty of Dentistry, University of Toronto, Toronto, ON, Canada; Institute of Neuroimmunology, Slovak Academy of Sciences, Bratislava, Slovakia

## Abstract

MirDIP is a well-established database that aggregates microRNA-gene human interactions from multiple databases to increase coverage, reduce bias, and improve usability by providing an integrated score proportional to the probability of the interaction occurring. In version 5.2, we removed eight outdated resources, added a new resource (miRNATIP), and ran five prediction algorithms for miRBase and mirGeneDB. In total, mirDIP 5.2 includes 46 364 047 predictions for 27 936 genes and 2734 microRNAs, making it the first database to provide interactions using data from mirGeneDB. Moreover, we curated and integrated 32 497 novel microRNAs from 14 publications to accelerate the use of these novel data. In this release, we also extend the content and functionality of mirDIP by associating contexts with microRNAs, genes, and microRNA–gene interactions. We collected and processed microRNA and gene expression data from 20 resources and acquired information on 330 tissue and disease contexts for 2657 microRNAs, 27 576 genes and 123 651 910 gene–microRNA–tissue interactions. Finally, we improved the usability of mirDIP by enabling the user to search the database using precursor IDs, and we integrated miRAnno, a network-based tool for identifying pathways linked to specific microRNAs. We also provide a mirDIP API to facilitate access to its integrated predictions. Updated mirDIP is available at https://ophid.utoronto.ca/mirDIP.

## INTRODUCTION

MicroRNAs (miRNAs) are short single stranded non-coding RNAs that play an essential role in gene regulation and thus are involved in a manifold of essential biological processes. In close interaction with Argonaute family proteins (AGO) (1), they form complex networks that regulate cell differentiation, development and homeostasis (2). The corresponding AGO–miRNA complexes are then guided to complementary (fully or partially) messenger RNAs (mRNAs) and can initiate regulatory mechanisms such as mRNA degradation, mRNA destabilization or mRNA deadenylation (3), as well as interference with mRNA translation (initiation, repression, elongation or termination) (4). While their regulatory impact on various biological processes, including development, cell growth and metabolism have long been known, several studies showed a crucial involvement in human pathologies, making them good candidates to become clinical biomarkers or therapeutic targets. For instance, a study by Hong *et al.* indicates regulatory effects on bone formation and regeneration as well as an involvement in inflammation, osteoporosis and periodontitis, and provides potential for gene therapeutic approaches involving miRNAs (5). A study by Kumar and Reddy revealed that the miR-455-3p expression level in people with Alzheimer's disease is significantly higher than in healthy individuals, and thus could be used as a diagnostic biomarker (6). MiRNAs can also be secreted, and exosomal miRNAs have been studied in different diseases. For example, lung exosomes are responsible for protective effects against stress signals and the maintenance of lung homeostasis. Air contamination and associated pulmonary diseases like asthma, however, may alter such composition thereby leading to dysregulation of exosomal miRNAs indicating a potential role as biomarker and therapeutic target in the pathogenesis of lung diseases (7). Similarly, miRNAs disrupted in a smoking status-dependent manner have been shown to affect lung cancer patient prognosis, representing biological markers for lung cancer prognosis or therapeutic intervention (8). Ultimately, miRNAs may also provide therapeutic targets for viral infections such as SARS-CoV-2 or HCV by interfering with the host-pathogen interactions (9).

These few examples demonstrate the importance of context annotation, and particularly tissue- and disease-specific miRNA expression analysis, which provide useful insights into their involvement in not only molecular and cellular processes, but more importantly, lead to identifying potential specific biomarkers and therapeutic targets (7). While a panacea of research and few databases have gathered information on the driving interactions amongst miRNAs and genes or gene products (10), no resources gather comprehensive context-specific miRNA information or annotate interactions with information such as tissue expression.

Similarly, it is important to keep the pace of the miRNAs discovery and annotation, to enable researchers to study the miRNA landscape with minimal biases and comprehensive annotation. While in the past miRBase was used as a reference database for miRNA research, in recent years the updates have become sparser, and some quality issues have started to arise (11). Nonetheless, miRBase is still considered the database of reference for miRNAs nomenclature.

The presented update of mirDIP aggregates miRNA-target interactions from multiple updated sources and subsequently annotates them with the previously described integrated score (12). MirDIP 5.2 also incorporates context annotation such as normal and disease tissues for miRNA-gene associations, which enables advanced analysis of condition-specific miRNA interaction networks. It also includes interaction predictions for miRBase and mirGeneDB miRNAs, expanding the number and the quality of miRNAs, as well as a curation of novel miRNAs from RNAseq studies in the literature.

## DATA COLLECTION

### Predicted interactions

To improve reliability and coverage, we have removed eight outdated resources (as they were not updated in over 10 years), and included miRNATIP (13). Moreover, we ran five algorithms using miRNAs from miRBase v.22 (14) and mirGeneDB 2.0 (15), and 3’ UTRs downloaded from Ensembl (May 2021, GRCh38 release 103): miranda (16), BiTargeting (17), PITA (18), RNAhybrid (19) and MirMAP (20). All the tools were run with default parameters, except for a threshold of –22 kcal/mol applied to RNAhybrid to filter interactions, as suggested in (21). More details about parameters used in each tool are listed in Supplementary Material, as well as which tools were excluded or failed to run. Any 3’-UTR sequences shorter than 25nt in length were not considered. Due to the overlap between mirGeneDB and miRBase, we ran only the miRNA sequences from mirGeneDB not linked to any miRBase ID (*n* = 78). For the resources downloaded, miRNA IDs were converted to miRBase v. 22 using miRBaseConverter (version 1.14.0 (22)) in R 4.0.3 (23) and gene symbols to HGNC May 2021 update using HGNC symbol checker (24). Supplementary Table S3 lists removed miRNA IDs. An integrated score was calculated as previously described (25), using the 24 resources described above, but our benchmark and validation set included the updated version of NPinter (v. 4) (26) and mirTarBase (v. 8) (27). Score classes are now assigned considering top percent targets per miRNA and not per entire mirDIP, providing a more homogeneous number of targets per miRNA, independently from the bias due to a miRNA being present in more resources (usually caused by a miRNA being included in miRBase at an earlier time). mirDIP 5.2 includes 46 364 047 predictions for 27 936 genes and 2734 miRNAs. mirDIP 5.2 is the only resource to provide interactions for the high-quality data from mirGeneDB.

### Novel miRNAs curation

A PubMed search was performed for ‘novel microRNA’ in Humans in December 2020, obtaining 210 papers. We considered only articles written in English, releasing human data and including genomic coordinates for each novel miRNA. We further explored papers not included in this search but cited by the remaining papers. We curated 14 papers and mapped all genomic coordinates to hg38 using the UCSC genome liftOver tool (http://genome.ucsc.edu/cgi-bin/hgLiftOver) when they were mapped to previous genomic releases. All curated manuscripts and the number of novel miRNAs collected are shown in Supplementary Table S1.

### MiRNA and gene context expression

To support users in analyzing miRNA and their interactions within the context of a specific tissue or disease, mirDIP 5.2 was extended with context information for miRNAs, genes, and corresponding miRNA-gene interactions. We obtained paired datasets from studies that analyzed both miRNA and mRNA expression of the same samples across multiple contexts, as listed in Tables 1 and 2. To reduce the issues related to the aging of array annotations, especially for miRNAs, we focused only on high-throughput micro- and mRNA sequencing datasets.

**Table 1.** Summary of miRNA tissue expression datasets used. * indicates datasets analyzed using Nextflow pipeline

PMID/GEO	Author	Number of miRNAs	Number of contexts
28108447^26^	Panwar	2562	23
29423032^31^	Naccarati	1765	3
29986767^32^	Schulze	2539	6
GSE134949	Rahman	2506	2
28877962^33^	McCall	2499	47
29625045^34^	TCGA	2563	72
34140680^35^	Lorenzi*	2656	192
34557219^36^	Varghese*	2656	2
33748479^37^	Vladimirova*	2656	1
31169949^38^	Ge*	2656	4
GSE149084	GeW*	2656	2
32759991^39^	Mao*	2656	2
31902369^40^	Hua*	2656	2
GSE181922	Francisco*	2656	2
All		2656	301

**Table 2.** Summary of gene tissue expression datasets used. * indicates datasets analyzed using Nextflow pipeline

PMID/GEO	Dataset	Number of genes	Number of contexts
26484569^31^	GTEx	18 340	55
30407591^27^	IID	17 313	46
29625045^32^	TCGA	19 847	72
29986767^33^	Schulze	22 461	2
34140680^34^	Lorenzi	21 723	206
34557219^35^	Varghese*	24 379	2
33748479^36^	Vladimirova*	24 379	1
31169949^37^	Ge*	24 379	4
GSE149084	GeW*	24 379	2
32759991^38^	Mao*	24 379	2
GSE137308	Lyu*	24 379	2
GSE126448	Bongiovanni*	24 379	1
23405175^39^	Kim*	24 379	2
**All**		**27 576**	**278**

To this aim, the Gene Expression Omnibus (GEO) (22) was queried on 3 March 2022 using the query:

‘Homo sapiens’[Organism] AND ‘Non-coding RNA profiling by high throughput sequencing’[Filter] AND ‘Expression profiling by high throughput sequencing’[Filter] AND ‘mirna-seq’[All Fields]

**Figure 1.**
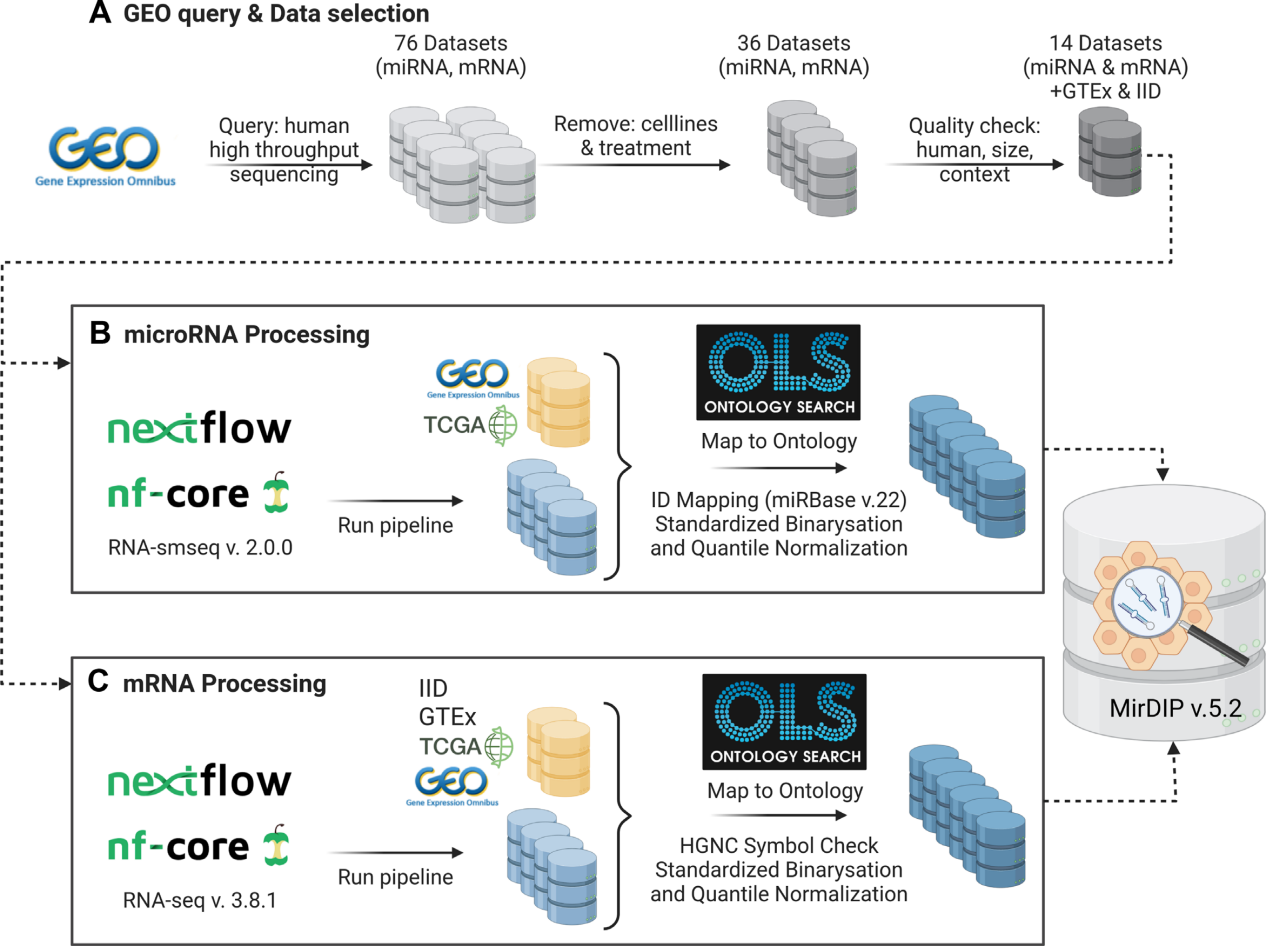
Overview of miRNA and gene expression dataset processing. Yellow icons refer to downloaded datasets, while blue icons to datasets processed in house. Created with BioRender.com

From GEO, we obtained 76 paired datasets that satisfied our search criteria (see Figure 1A). In the subsequent selection process, 40 paired datasets were excluded because the studies focused only on cells, cell lines, or treatment. Of the remaining paired datasets, 22 were excluded due to a lack of available data, non-human samples, or included too few patients or context that did not map to a disease or tissue ontology (e.g. ‘*in vitro* fertilization’). 14 remaining paired datasets were submitted to the Nextflow pipelines for processing, see Figures 1B and C, respectively. The raw data sets were processed using the Nextflow pipeline RNA-seq v. 3.8.1 (https://nf-co.re/rnaseq/3.8.1) for RNA-seq data (Figure 1C) and smRNA-seq v. 2.0.0 (https://nf-co.re/smrnaseq) for miRNA-seq data (Figure 1B) on a server running the CentOS 7 operating system with 40 threads with hyperthreading available on the 20 cores. Details on packages and versions used for processing in these pipelines is described in Supplementary Material. Eight of the 14 datasets were successfully processed in smRNA-seq and five of the 14 corresponding mRNAs in mRNA-seq, while the remaining failed to run. In parallel, we collected data from datasets that included multiple tissues, even if they studied only miRNAs. These datasets were derived from other databases of miRNA tissue expression. More details on all the datasets are available in Supplementary Table S2.

For the datasets not run through the Nextflow pipeline, pre-processed data were downloaded from GEO, except for:

- The Panwar dataset was obtained through its R package miRmine (v1.12.0) (28).
- TCGA (clinical, mRNA, and miRNA) was downloaded from https://gdac.broadinstitute.org.
- GTEx was downloaded from https://www.gtexportal.org/home/datasets.
- IID has been obtained from http://ophid.utoronto.ca/iid (29).

All miRNA datasets used to extract context information included in mirDIP v.5.2 are listed in Table 2. Each dataset was post-processed separately to ensure that all miRNAs were updated to miRBase v.22 IDs and that all tissue/cell type/disease names were consistent across the datasets. miRNA identifiers were updated using miRBaseConverter (v1.14.0) in R 4.0.3 when possible; otherwise, they were removed (Supplementary Table S4). Moreover, all gene expression datasets, as listed in Table 2, were post-processed to ensure that all gene symbols were consistent with the HGNC-approved symbols (https://www.genenames.org/tools/multi-symbol-checker/). To ensure term consistency, we used the Disease Ontology (30) and BRENDA Tissue Ontology (31) to standardize context names. Out of the 1250 relationships present in the created ontology, 53% were obtained through these two ontologies. When a term was not present, its relationships were identified through other ontologies in the Ontology Lookup Service (OLS)—namely FMA (http://si.washington.edu/projects/fma), NCIT (https://github.com/NCI-Thesaurus/thesaurus-obo-edition), UBERON (32) and OBA (https://github.com/obophenotype/bio-attribute-ontology), accounting for 20% of the relationships. Finally, we curated the remaining 27% of relationships to map them to terms already included. ACH, ZA, GKAE mapped the terms and CP verified them. Contexts corresponding to cell lines and qualifiers outside normal and disease (for example, developmental stage) were not included in this release.

All miRNA expression information was converted into binary values. For smRNA-Seq datasets, a miRNA was considered ‘expressed’ in a context if any of the replicates for the context had the miRNA expressed (i.e. had a non-zero expression value). Moreover, to enable a more fine-grained analysis of miRNA abundance, miRNA expression values were quantile-normalized (see mirDIP-Tissues (scale)). Therefore, for each sample, any miRNA with an expression value of zero remained so. The remaining non-zero values were converted to a number between one and five that represented which of the 20th percentiles of non-zero values it corresponded to. The percentiles were taken from the non-zero values in order to account for the varying fraction of zero values (sometimes up to 80%) among the samples. Then for each context, its quantile-normalized values were averaged per miRNA (biological replicates, for instance). Any miRNA ID-sequence pair that appeared multiple times in a dataset (as a result of redundancy in precursor-to-mature ID mappings) was merged prior to transformation. Likewise, the gene expression datasets were post-processed and transformed to binary values in the same procedure as previously described for miRNA expression. The datasets are listed in Table 2.

Finally, miRNA and gene expression data and the associated context information were integrated into the mirDIP database. To support variety of workflows, mirDIP supports multiple types of queries:

1. The Search Tissues via miRNAs and miRNAs (scale) option enables the user to directly search for binary or quantile-normalized miRNA context association, respectively. The information from the selected datasets is combined to give a score for each miRNA in each context that represents how well supported the miRNA’s expression is in that context. For a query on binary values, the score of a miRNA in a context is the number of selected sources in which the miRNA is expressed in the context divided by the number of selected sources that measure the miRNA in the context.
2. The Search Tissues and interactions option integrates both miRNA and gene contexts with the corresponding mirDIP interaction information and thus selects context-specific interactions.
3. The Tissue Matrix option allows querying of both context-specific miRNA and interactions and presents the results in an accumulated matrix that contains one column per tissue.

For each miRNA-gene interaction in the mirDIP database, the context is scored based on information about both miRNA and gene expression being measured across multiple datasets. A value of 1 indicates that both entities’ expression is unanimous among their respective sources (i.e. all the datasets that measured the gene and miRNA in that context), while value 0 means that at least one of the two molecules has no expression in the context amongst all its sources.

**Figure 2.**
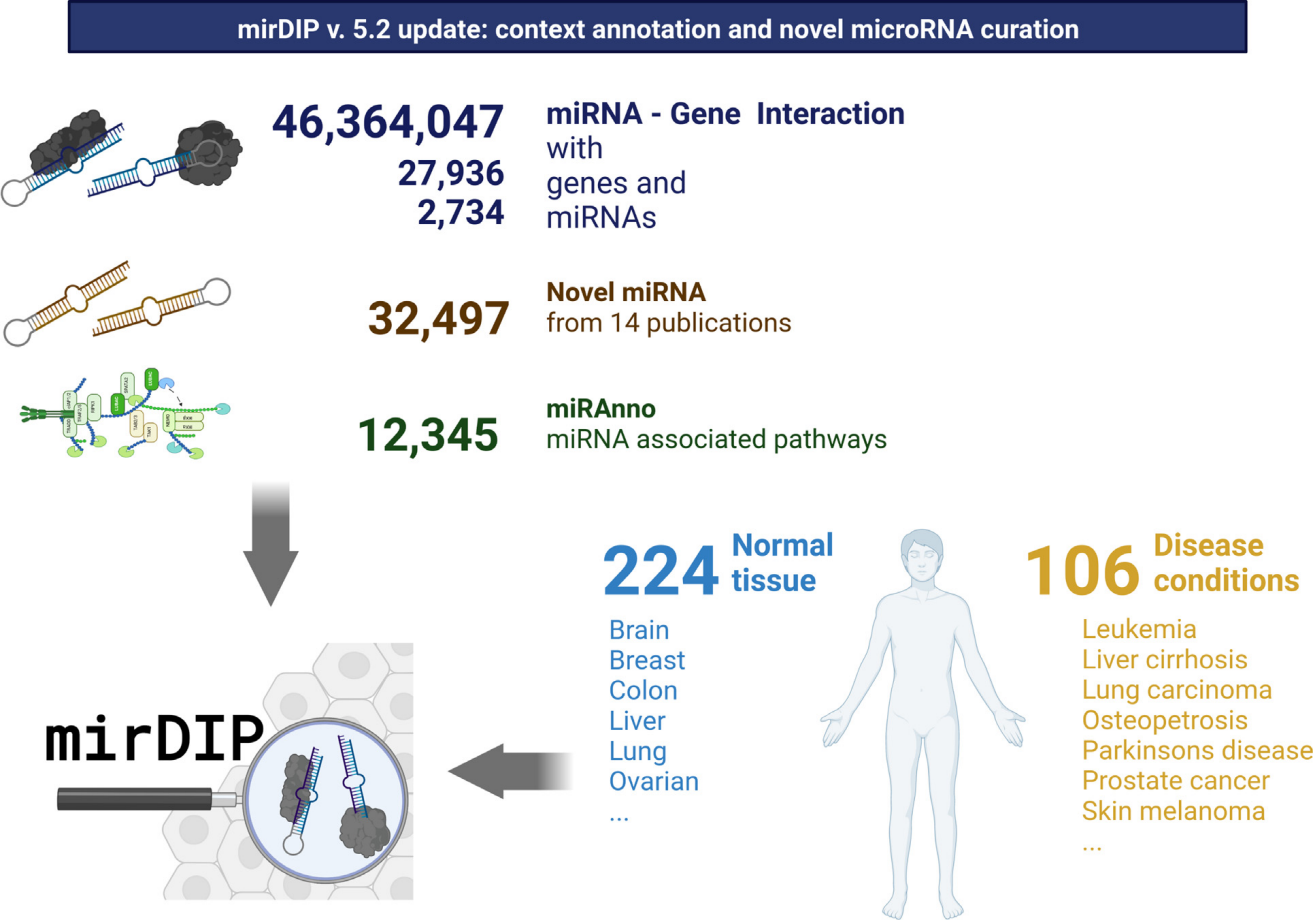
Graphical overview of mirDIP v5.2 content, context annotation and novel miRNA curation. Created with BioRender.com.

Figure 2 summarizes data collection.

## DATABASE CONTENT

### Novel features

Several functionalities have been added to the database: a researcher can now input precursor IDs and retrieve predicted interactions for their corresponding mature miRNAs. This is particularly useful due to the increasing amount of high-throughput data providing precursor-based data (and the lack of online tools to translate a precursor ID to its mature counterparts).

mirDIP 5.2 includes an API that allows users to query the database programmatically using R, Python or Java, to transform the data as needed and into their favorite format, and to include the results in their pipelines for further analyses. The database also integrates miRAnno (33), a tool that measures the association between miRNAs and individual pathways. mirDIP data can be downloaded as tab-separated text files, FASTA and GFF. The ontology network can be downloaded in NAViGaTOR n4n format.

### Updated content: improved coverage

#### Novel miRNAs

**Figure 3.**
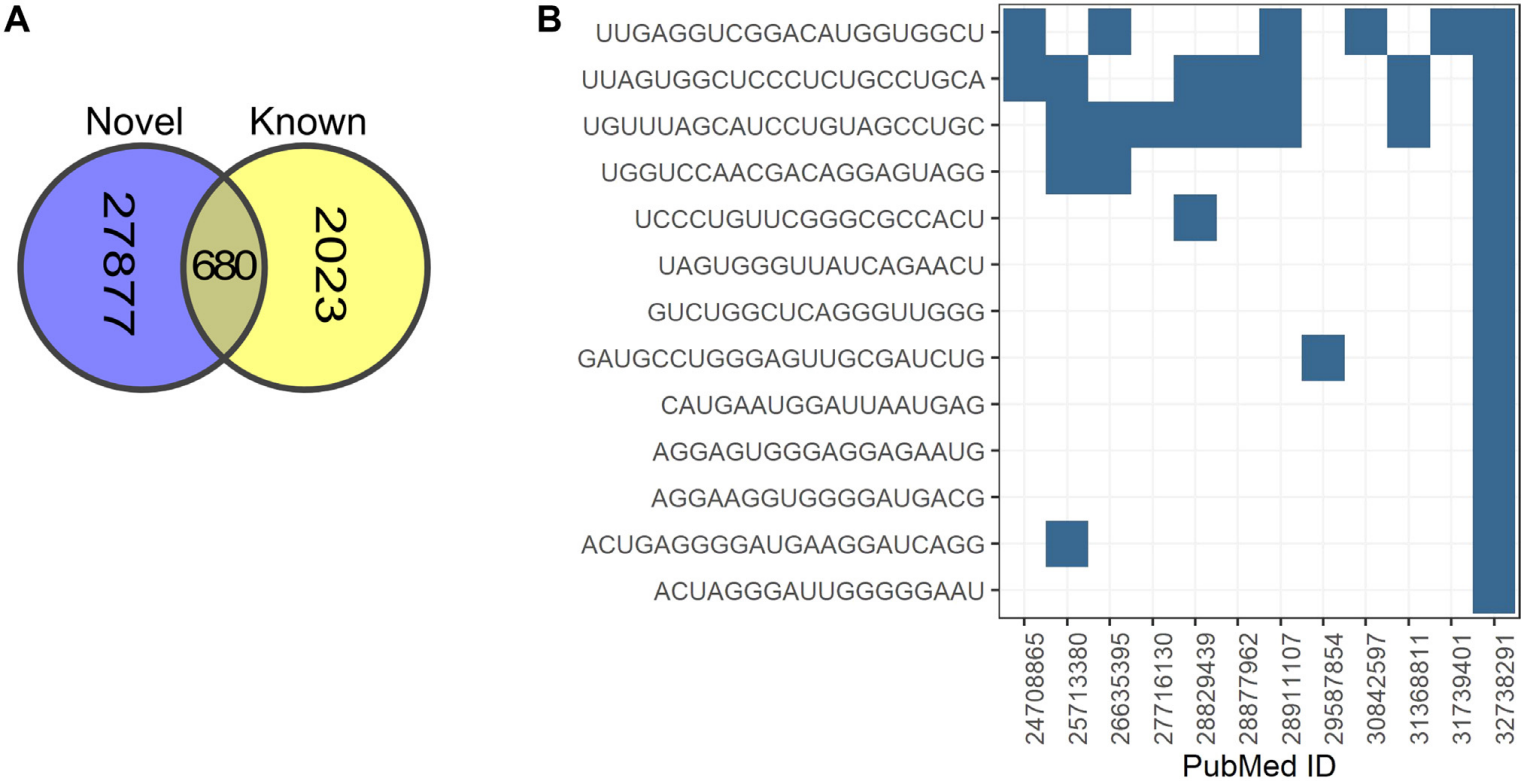
(**A**) Overlap between unique novel miRNA sequences and sequences of known miRNAs (derived from miRBase or mirGeneDB). (**B**). Overlap between novel miRNA sequences in Ali et al. and sequences of novel miRNAs in other curated papers.

There is currently no resource that systematically collects, annotates, and makes available RNAseq data for discovering novel, often tissue-specific miRNAs. miRCarta (34) allows the search for overlap of a miRNA sequence to miRNAs that users analyzed in miRMaster or that were published in (35) and (36), but does not allow users to gather all miRNAs present in one publication (or miRMaster dataset). As miRBase no longer provides regular new releases, the gap between the set of curated miRNAs and more recently discovered novel miRNAs is increasing. To address this gap, we curated and collected 32 497 novel miRNAs (28 557 unique sequences) from 14 publications and provide the user with the possibility to search for specific sequences or complete studies. We also provide sequence overlap among novel miRNAs and between novel and known miRNAs (both from miRBase and mirGeneDB). 680 of the novel miRNA sequences are already present in either one of the databases of known miRNAs (Figure 3A).

For example, a study by Ali *et al.* (37) identified 13 novel miRNAs. The authors looked for overlap between their novel miRNAs and miRNAs identified by Londin *et al.* (35), and 4 overlapping miRNAs were identified. Looking for the sequences of the same 13 miRNAs in mirDIP, it is now possible to quickly identify the same overlap across multiple studies. In this case, 7 overlapping miRNAs were identified, 3 of which are present in 5, 6 and 7 other papers, as shown in Figure 3B, making these miRNAs quite interesting for further curation in databases like miRbase, and prioritization for research.

### Novel content: added biological context

#### miRNA and gene context

Scored miRNA–gene predictions are fundamental to help researchers to prioritize interactions to validate, as well as to identify molecular functions linked to the sets of gene targets for specific miRNAs of interest. Nonetheless, not all miRNAs are expressed equally across diverse tissues (similar to all other molecules, from genes to proteins to other non-coding RNAs). To address this, we collected miRNA expression for 301 tissue/cell type and disease contexts, with 209 different normal tissues and cell types (at varying levels of tissue specificity) and 92 different disease conditions. Combined, mirDIP v.5.2 tissue annotation includes 2656 miRNA IDs, that can be queried both as binary (presence/absence) or as scaled classes of expression, see Table 1 for details on the data origin.

**Figure 4.**
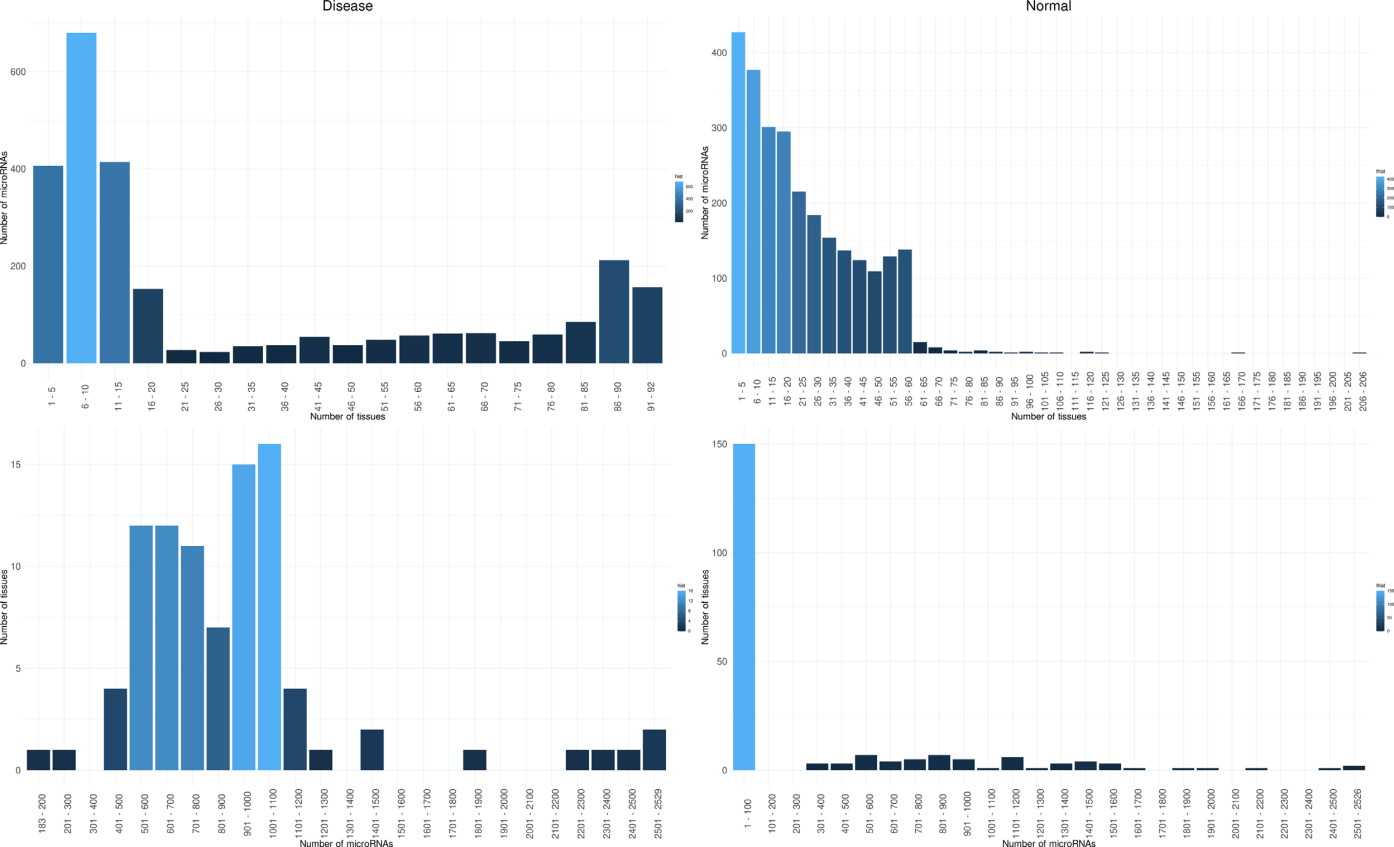
Number of miRNAs expressed in a set of tissues (top, **A** and **B**) and number of tissues per set of miRNAs (bottom, **C** and **D**) in normal tissues (right) and disease conditions (left). The plot was obtained using only canonical miRNAs and binary values.

Figure 4 shows the distribution of miRNAs expressed per tissue or disease and the number of tissues or diseases where a set of miRNAs is expressed, for canonical miRNAs. Interestingly, in disease conditions miRNAs appear tissue specific, and only a few miRNAs are expressed across multiple tissues, while in normal tissues more miRNAs are expressed across tissues. Moreover, mirDIP v.5.2 integrates gene expression for 278 tissue/cell type and disease contexts, comprising 188 different normal tissues and cell types (at varying levels of tissue specificity) and 90 different disease conditions for 27 576 genes.

In disease context, miRNAs are expressed in as little as 1 to a maximum of 92 contexts, while in normal tissues the range expands from 1 to 206. Conversely, while in disease the different contexts express a number of microRNAs that varies from 183 to 2529 miRNAs, in normal it varies from 1 to 2526.

**Figure 5.**
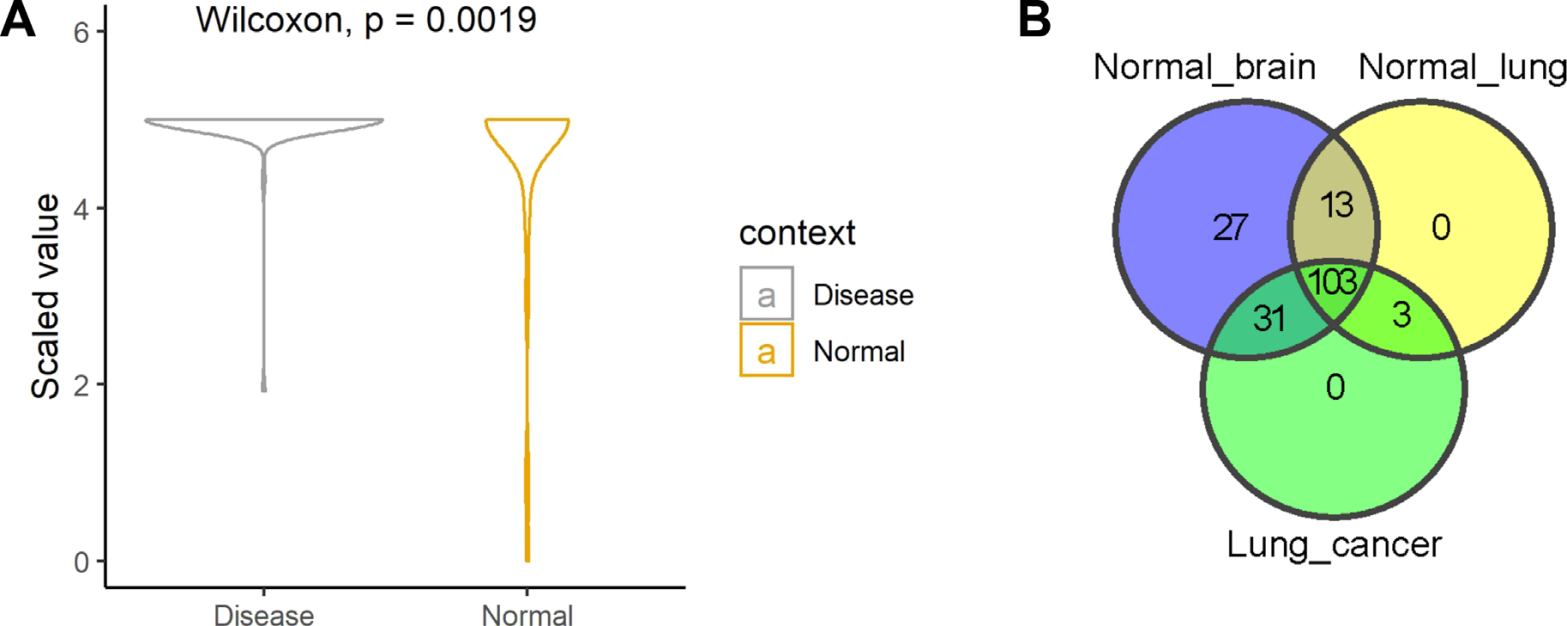
(**A**) Classes of expression for hsa-miR-92a-3p in disease conditions and normal tissues. (**B**). Overlap of expressed miRNAs among normal brain, normal lung and lung cancer.

For example, hsa-miR-92a-3p is the miRNA expressed both in most normal tissues (237) and across disease conditions (93), and it has notably been linked to multiple different diseases (90 according to HMDD (38)), suggesting that, rather than its presence/absence, smaller changes in its expression level could have a dramatic effect on the miRNA’s downstream effectors. Looking at the classes of expression for this miRNA, it is apparent that in disease conditions, the miRNA tends to be less expressed than in normal tissues (Figure 5A).

Akin to miRNA context-specific expression, it is important to know if the miRNA’s target gene is expressed in the relevant tissue or disease. For example, a researcher could be interested in investigating whether specific miRNAs could be involved in brain metastasis due to lung cancer. Hypothesizing that the development of the brain metastasis is due to the interaction between the metastatic cancer cells and normal brain environment (39), a mirDIP search would focus on miRNAs expressed in lung cancer and normal brain but not in normal lung. Searching for miRNAs expressed in such conditions, the researcher can see that 31 miRNAs are common between lung cancer and normal brain, but not expressed in normal lung (Figure 5B). Using a ‘very high’ threshold, 3,951 genes are found to be targeted by these 31 miRNAs. Two genes (MECP2 and ONECUT2) are targeted by 9 out of 31 miRNAs, while the remaining genes are targeted by fewer miRNAs. MECP2 is a transcriptional regulator that is frequently amplified as an oncogene in many cancers, including lung cancer (40). It has been demonstrated that this overexpression supports invasion and metastasis through SPI1 and ZEB1 (41). Moreover, MECP2 is well known for its role in regulating postnatal brain development, and its involvement in several neurodevelopmental disorders (42). Looking at the interactions between the miRNAs and MECP2, the researcher can see that all 9 interactions are common between lung cancer and normal brain, providing an interesting set to further explore in the context of brain metastasis from lung cancer, with a specific computational model to use as a guide.

## CONCLUSION

mirDIP 5.2 provides increased coverage, and richer annotation of, miRNA-target predictions. Our predictions include data precalculated from databases as well as data run in house using prediction specific algorithms. The tools publication or update span different years (as shown in Supplementary Table S5), and we tried to include data from more classic but well-maintained tools to databases using more recent methods (well reviewed in (43)). Database usability is improved by enabling queries with precursor IDs, providing tissue- and disease-specific miRNA-gene target associations, supporting analytical workflows through an API, and providing more accurate miRNA-pathway annotation with miRAnno.

The inclusion of high-quality known data from mirGeneDB and curated data from published novel miRNAs provides different types of miRNAs, enabling researchers to answer broader types of questions with less bias. The integration of tissue and disease expression annotations for miRNAs and their interactions enables biologically relevant translational research.

## DATA AVAILABILITY

mirDIP 5.2 is publicly available at https://ophid.utoronto.ca/mirDIP. Code to replicate our data is available at https://github.com/ijlab/mirdip.

## Supplementary Material

gkac1070_Supplemental_File
