# Supplementary material for: MirDIP 5.2: tissue context annotation and novel microRNA curation
Source: Nucleic Acids Res. 2022 Dec 1;51(D1):D217–25. doi: 10.1093/nar/gkac1070 (PMC9825511; doi:10.1093/nar/gkac1070)
Supplement: gkac1070_Supplemental_File [file gkac1070_supplemental_file.pdf]

Supplementary Table 1. Novel miRNAs in each curated publication

PubMed ID (Ref)	Number of novel miRNAs
28829439[1]	13,088
28911107[2]	6,090
25713380[3]	3,707
34140680[4]	3,648
24708865[5]	2,469
26635395[6]	1,036
28877962[7]	990
30842597[8]	468
31739401[9]	340
31828039[10]	292
31368811[11]	154
29587854[12]	103
27716130[13]	99
32738291[14]	13

1. de Rie, D. *et al.* An integrated expression atlas of miRNAs and their promoters in human and mouse. *Nat Biotechnol* **35**, 872–878 (2017).
2. Fehlmann, T. *et al.* Web-based NGS data analysis using miRMaster: a large-scale meta-analysis of human miRNAs. *Nucleic Acids Res* **45**, 8731–8744 (2017).
3. Londin, E. *et al.* Analysis of 13 cell types reveals evidence for the expression of numerous novel primate- and tissue-specific microRNAs. *Proc Natl Acad Sci U S A* **112**, E1106-15 (2015).
4. Lorenzi, L. *et al.* The RNA Atlas expands the catalog of human non-coding RNAs. *Nat Biotechnol* **39**, 1453–1465 (2021).
5. Friedländer, M. R. *et al.* Evidence for the biogenesis of more than 1,000 novel human microRNAs. *Genome Biol* **15**, (2014).
6. Backes, C. *et al.* Prioritizing and selecting likely novel miRNAs from NGS data. *Nucleic Acids Res* **44**, (2016).
7. McCall, M. N. *et al.* Toward the human cellular microRNAome. *Genome Res* **27**, 1769–1781 (2017).
8. Barros-Filho, M. C. *et al.* Previously undescribed thyroid-specific miRNA sequences in papillary thyroid carcinoma. *J Hum Genet* **64**, 505–508 (2019).

9. Pewarchuk, M. E. *et al.* Upgrading the Repertoire of miRNAs in Gastric Adenocarcinoma to Provide a New Resource for Biomarker Discovery. *Int J Mol Sci* **20**, (2019).
10. Rock, L. D. *et al.* Expanding the Transcriptome of Head and Neck Squamous Cell Carcinoma Through Novel MicroRNA Discovery. *Front Oncol* **9**, (2019).
11. Martinez, V. D. *et al.* Discovery of Previously Undetected MicroRNAs in Mesothelioma and Their Use as Tissue-of-Origin Markers. *Am J Respir Cell Mol Biol* **61**, 266–268 (2019).
12. Minatel, B. C. *et al.* Large-scale discovery of previously undetected microRNAs specific to human liver. *Hum Genomics* **12**, (2018).
13. Wake, C. *et al.* Novel microRNA discovery using small RNA sequencing in post-mortem human brain. *BMC Genomics* **17**, (2016).
14. Ali, S. A. *et al.* Sequencing identifies a distinct signature of circulating microRNAs in early radiographic knee osteoarthritis. *Osteoarthritis Cartilage* **28**, 1471–1481 (2020).

Supplementary Table 2. Processed datasets for tissue annotation

Dataset name	Link	Molecule	Date of Download	Notes
Panwar	http://bioconductor.org/packages/release/bioc/html/miRmine.html	microRNA	June 13th 2021	
Naccarati	GSE128359	microRNA	July 28th 2021	
Schulze	GSE110719	microRNA	July 28th 2021	
Rahman	GSE134949	microRNA	July 28th 2021	
McCall	https://genome.cshlp.org/content/early/2017/09/06/gr.222067.117	microRNA	July 16th 2021	Supplementary Table 5
Lorenzi*	GSE138734	microRNA	June 10th 2022	
Varghese*	GSE176288	microRNA	May 13th 2022	
Vladimirova*	GSE138042	microRNA	May 13th 2022	
Ge*	GSE121842	microRNA	May 12th 2022	
GeW*	GSE149084	microRNA	May 13th 2022	
Mao*	GSE138518	microRNA	May 16th 2022	
Hua*	GSE135055	microRNA	May 13th 2022	
Francisco*	GSE181922	microRNA	May 15th 2022	
TCGA	https://gdac.broadinstitute.org/	microRNA	Aug 30th 2022	Used processed data

GTEx	GTEx portal	mRNA	June 10th 2021	
IID	IID website	mRNA	May 24th 2021	
Lorenzi	GSE138734	mRNA	Aug 30th 2022	Used processed data
Varghese*	GSE176271	mRNA	May 24th 2022	
Vladimirova*	GSE138042	mRNA	May 4th 2022	
Ge*	GSE121842	mRNA	May 10th 2022	
GeW*	GSE149084	mRNA	May 4th 2022	
Mao*	GSE138518	mRNA	May 12th 2022	
Lyu*	GSE137308	mRNA	May 13th 2022	
Bongiovanni*	GSE126448	mRNA	May 16th 2022	
Kim*	GSE37765	mRNA	May 25th 2022	
Schulze	GSE110719	mRNA	Aug 30th 2022	Used processed data
TCGA	https://gdac.broadinstitute.org/	mRNA	Aug 30th 2022	Used processed data

* Datasets run through Nextflow pipeline

ID conversions

3' UTR sequences corresponding to GRCh38 release 103 were downloaded from Ensembl on May 17th, 2021 using the BioMart web interface (http://useast.ensembl.org/biomart/martview/dbb0ff13a8229396fa5e492173880654). Any 3'-UTR sequences shorter than 25nt in length were not considered, and the unique list of ensembl gene identifiers remaining was mapped to the latest HGNC via both Ensembl (downloaded May 26th 2021 from BioMart) and HGNC publicly available mapping files downloaded from http://ftp.ebi.ac.uk/pub/databases/genenames/hgnc/tsv/hgnc_complete_set.txt on May 19 th, 2021. If there was no mapping, we discarded the 3'-UTR sequence in question from the dataset to avoid ambiguity.

The same process was applied to downloaded datasets, if they published Ensembl identifiers. In the case that a source dataset published RefSeq identifiers, the HGNC mapping from RefSeq to HGNC gene names, which is present in the aforementioned files downloaded from HGNC, was used to standardize identifiers, and any identifiers that could not be standardized to HGNC were discarded.

In the case of datasets publishing HGNC gene symbols directly or datasets published in mirDIP version 4.1, a unique list of all such identifiers was input into the HGNC symbol checker tool (https://www.genenames.org/tools/multi-symbol-checker/). Where the input symbol was noted by HGNC as a “Previous symbol” or “Alias symbol”, the input symbol was updated to the value in the “Approved symbol” column in mirDIP version 5.2, and all cases where the input symbol was noted as “Unmatched” or “Withdrawn symbol” resulted in records being discarded from the dataset.

Details of run tools

Tool	Version	Default	Notes
Bi-Targeting	2010	Yes	Run with options:<br>max_err = 6<br>window_size = 30<br>GU = true<br>seed_start =<br>2<br>seed_end = 8<br>max_miRNA_bulge =<br>6<br>max_gene_bulge = 6<br>max_GU_in_seed =<br>1<br>max_GU_total = 4<br>energy_filter =<br>false<br>energy_cutoff = -15<br>energy_normalized_cutoff = 0.4
miRanda	3.3a	Yes	
mirMAP	1.1	Yes	
PITA	6.0	Yes	Filtered for energy <= -13.0 kcal/mol
RNAhybrid	2.1.2		Filtered for energy <= -22.0 kcal/mol, and p-value <= 0.1'

Nextflow details

Nextflow version 21.10.6.5660 runs on a private cloud with 12 bare metal x86_64 architecture servers running the CentOS 7 operating system, kernel version 3.10.0-514.el7.x86_64. Each server is identically configured, featuring the Intel(R) Xeon(R) CPU E5-2660 v3 @ 2.60GHz CPU, which equates to 40 threads with hyperthreading available on the 20 cores per CPU. Each server has 64GB of memory, some local storage for temporary files, and are connected to the GPFS storage area network by a 10GBit network fabric. Jobs on a cluster are run using the SLURM job scheduling framework.

Nextflow tools versions

RNA-seq pipeline:

- bbmap:38.93--he522d1c_0
- bedtools:2.30.0--hc088bd4_0
- bioconductor-dupradar:1.18.0--r40_1
- bioconductor-summarizedexperiment:1.20.0--r40_0
- bioconductor-tximeta:1.8.0--r40_0
- hisat2:2.2.1--h1b792b2_3
- multiqc:1.11--pyhdfd78af_0
- perl:5.26.2
- picard:2.26.10--hdfd78af_0
- preseq:3.1.2--h445547b_2
- python:3.9--1
- qualimap:2.2.2d--1
- rseqc:3.0.1--py37h516909a_1
- salmon:1.5.2--h84f40af_0
- samtools:1.15.1--h1170115_0
- stringtie:2.2.1--hecb563c_2
- subread:2.0.1--hed695b0_0
- ucsc-bedclip:377--h0b8a92a_2
- ucsc-bedgraphtobigwig:377--h446ed27_1
- Umi_tools:1.1.2--py38h4a8c8d9_0

Code to run it

```
nextflow run rnaseq --genome GRCh38 --input /samplesheet.csv --star_index
false --gene_bed false --aligner star_rsem --outdir /outputdirectory --
save_merged_fastq -profile ijcluster
```

smRNA-seq:

- fastqc:0.11.9--0
- biocontainers:v1.2.0_cv1
- bioconvert:0.4.3--py_0
- bowtie:1.3.0--py38hcf49a77_2
- fastx_toolkit:0.0.14--he1b5a44_8
- mirdeep2:2.0.1.3--hdfd78af_1
- mirtrace:1.0.1--hdfd78af_1
- multiqc:1.11--pyhdfd78af_0
- python:3.8.3
- r-data.table:1.12.2
- samtools:1.14--hb421002_0
- seqcluster:1.2.8--pyh5e36f6f_0
- seqkit:2.0.0--h9ee0642_0
- Trim-galore:0.6.7--hdfd78af_0

Code to run it:

```
nextflow run nf-core/smrnaseq -profile ijcluster --input /samplesheet.csv
--outdir /outputdirectory --genome GRCh38 --protocol qiaseq --
mirtrace_species hsa -r gittak_ac_config
```

Supplementary Table 3

**MicroRNAs excluded due to ID conversion**	**Tools excluding such MicroRNAs**
hsa-miR-1254	BCmicro,BiTargeting,CoMeTa,Cupid,DIANA,ElMMo3,MBStar,miRancestarPredictions,miRDB,MirMAP,mirnatip,MirSNPInTarget,miRTar2GO,mirzag,PACCMIT,reptar,RNA22,RNAhybrid,TargetRank,TargetSpy
hsa-miR-1273a	BCmicro,BiTargeting,CoMeTa,Cupid,DIANA,ElMMo3,MBStar,microrna,miRancestarPredictions,miRDB,MirMAP,mirnatip,MirSNPInTarget,MirTar2,mirzag,PACCMIT,reptar,RNA22,RNAhybrid,TargetRank,TargetSpy

hsa-miR-566	BiTargeting,CoMeTa,Cupid,DIANA,ElMMo3,MBStar,microrna,miRancestarPredictions,mirbase,miRDB,MirMAP,mirnatip,MirSNPInTarget,MirTar2,mirzag,MultiMiTar,PACCMIT,reptar,RNA22,RNAhybrid,TargetRank,TargetSpy
hsa-miR-3653-3p	BiTargeting,Cupid,DIANA,ElMMo3,MBStar,miRancestarPredictions,miRDB,MirMAP,mirnatip,MirSNPInTarget,MirTar2,PACCMIT,RNA22,RNAhybrid,TargetRank,TargetSpy
hsa-miR-3607-5p	BiTargeting,Cupid,DIANA,ElMMo3,MBStar,miRancestarPredictions,miRDB,MirMAP,mirnatip,MirSNPInTarget,MirTar2,mirzag,PACCMIT,RNA22,RNAhybrid,TargetRank,TargetSpy
hsa-miR-3653	BiTargeting,Cupid,DIANA,ElMMo3,MBStar,miRancestarPredictions,miRDB,MirMAP,mirnatip,MirSNPInTarget,MirTar2,mirzag,PACCMIT,RNA22,RNAhybrid,TargetRank,TargetSpy
hsa-miR-1273e	BiTargeting,Cupid,DIANA,ElMMo3,MBStar,miRancestarPredictions,miRDB,MirMAP,mirnatip,MirSNPInTarget,MirTar2,mirzag,PACCMIT,RNA22,RNAhybrid,TargetRank,TargetSpy
hsa-miR-3607-3p	BiTargeting,Cupid,DIANA,ElMMo3,MBStar,miRancestarPredictions,miRDB,MirMAP,mirnatip,MirSNPInTarget,MirTar2,mirzag,PACCMIT,RNA22,RNAhybrid,TargetRank,TargetSpy
hsa-miR-3687	BiTargeting,Cupid,DIANA,ElMMo3,MBStar,miRancestarPredictions,miRDB,MirMAP,mirnatip,MirSNPInTarget,MirTar2,mirzag,PACCMIT,RNA22,RNAhybrid,TargetRank,TargetSpy
hsa-miR-1273d	BiTargeting,Cupid,DIANA,ElMMo3,MBStar,microrna,miRancestarPredictions,miRDB,MirMAP,mirnatip,MirSNPInTarget,MirTar2,mirzag,PACCMIT,reptar,RNA22,RNAhybrid,TargetSpy
hsa-miR-3656	BiTargeting,DIANA,ElMMo3,MBStar,miRancestarPredictions,miRDB,MirMAP,mirnatip,MirSNPInTarget,MirTar2,mirzag,PACCMIT,RNA22,RNAhybrid,TargetRank,TargetSpy
hsa-miR-4532	BiTargeting,DIANA,MBStar,miRancestarPredictions,miRDB,MirMAP,mirnatip,MirSNPInTarget,MirTar2,mirzag,PACCMIT,RNA22,RNAhybrid,TargetRank,TargetSpy
hsa-miR-4792	BiTargeting,DIANA,MBStar,miRancestarPredictions,miRDB,MirMAP,mirnatip,MirSNPInTarget,MirTar2,mirzag,PACCMIT,RNA22,RNAhybrid,TargetRank,TargetSpy

hsa-miR-4419a	BiTargeting,DIANA,MBStar,miRancestarPredictions,miRDB,MirMAP,mirnatip,MirSNPInTarget,MirTar2,mirzag,PACCMIT,RNA22,RNAhybrid,TargetRank,TargetSpy
hsa-miR-4461	BiTargeting,DIANA,MBStar,miRancestarPredictions,miRDB,MirMAP,mirnatip,MirSNPInTarget,MirTar2,mirzag,PACCMIT,RNA22,RNAhybrid,TargetRank,TargetSpy
hsa-miR-4417	BiTargeting,DIANA,MBStar,miRancestarPredictions,miRDB,MirMAP,mirnatip,MirSNPInTarget,MirTar2,mirzag,PACCMIT,RNA22,RNAhybrid,TargetRank,TargetSpy
hsa-miR-4419b	BiTargeting,DIANA,MBStar,miRancestarPredictions,miRDB,MirMAP,mirnatip,MirSNPInTarget,MirTar2,mirzag,PACCMIT,RNA22,RNAhybrid,TargetRank,TargetSpy
hsa-miR-1273g-5p	BiTargeting,DIANA,MBStar,miRancestarPredictions,miRDB,MirMAP,mirnatip,MirSNPInTarget,MirTar2,mirzag,PACCMIT,RNA22,RNAhybrid,TargetRank,TargetSpy
hsa-miR-1273g-3p	BiTargeting,DIANA,MBStar,miRancestarPredictions,miRDB,MirMAP,mirnatip,MirSNPInTarget,MirTar2,mirzag,PACCMIT,RNA22,RNAhybrid,TargetRank,TargetSpy
hsa-miR-1273f	BiTargeting,DIANA,MBStar,miRancestarPredictions,miRDB,MirMAP,mirnatip,MirSNPInTarget,MirTar2,mirzag,PACCMIT,RNA22,RNAhybrid,TargetRank,TargetSpy
hsa-miR-5096	BiTargeting,DIANA,MBStar,miRancestarPredictions,miRDB,MirMAP,mirnatip,MirSNPInTarget,MirTar2,mirzag,PACCMIT,RNA22,RNAhybrid,TargetRank,TargetSpy
hsa-miR-4459	BiTargeting,DIANA,MBStar,miRancestarPredictions,miRDB,MirMAP,mirnatip,MirSNPInTarget,MirTar2,mirzag,PACCMIT,RNA22,RNAhybrid,TargetRank,TargetSpy
hsa-miR-5095	BiTargeting,DIANA,MBStar,miRancestarPredictions,miRDB,MirMAP,mirnatip,MirSNPInTarget,MirTar2,mirzag,PACCMIT,RNA22,RNAhybrid,TargetRank,TargetSpy
hsa-miR-6087	BiTargeting,MBStar,miRancestarPredictions,miRDB,MirMAP,mirnatip,mirzag,RNA22,RNAhybrid,TargetSpy
hsa-miR-6723-5p	BiTargeting,MBStar,miRancestarPredictions,miRDB,MirMAP,mirnatip,mirzag,RNA22,RNAhybrid,TargetSpy
hsa-miR-7641	BiTargeting,miRancestarPredictions,miRDB,mirnatip,mirzag,RNA22,RNAhybrid,TargetSpy
hsa-miR-3669	mirzag
hsa-miR-3673	mirzag
hsa-miR-4520b-5p	mirzag
hsa-miR-455-3p.1	targetscan

hsa-miR-455-3p.2	targetscan
hsa-miR-483-3p.2	targetscan
hsa-miR-496.1	targetscan
hsa-miR-496.2	targetscan
hsa-miR-504-5p.1	targetscan
hsa-miR-505-3p.1	targetscan
hsa-miR-505-3p.2	targetscan
hsa-miR-483-3p.1	targetscan
hsa-miR-101-3p.1	targetscan
hsa-miR-411-5p.1	targetscan
hsa-miR-101-3p.2	targetscan
hsa-miR-124-3p.1	targetscan
hsa-miR-124-3p.2	targetscan
hsa-miR-126-3p.1	targetscan
hsa-miR-126-3p.2	targetscan
hsa-miR-133a-3p.1	targetscan
hsa-miR-133a-3p.2	targetscan
hsa-miR-140-3p.1	targetscan
hsa-miR-140-3p.2	targetscan
hsa-miR-142-3p.1	targetscan
hsa-miR-142-3p.2	targetscan
hsa-miR-183-5p.1	targetscan
hsa-miR-183-5p.2	targetscan
hsa-miR-203a-3p.1	targetscan
hsa-miR-203a-3p.2	targetscan
hsa-miR-302c-3p.1	targetscan

hsa-miR-302c-3p.2	targetscan
hsa-miR-325-3p	targetscan
hsa-miR-873-5p.1	targetscan
hsa-miR-383-5p.1	targetscan
hsa-miR-383-5p.2	targetscan

Supplementary Table 4

Dataset name	Number of MicroRNAs that were excluded due to ID conversion
Panwar	266
Naccarati	1,765
Rahman	380
McCall	47

Supplementary Table 5

Database	last update	
miRDB	2019	
TargetScan	2018	
miRTar2GO	2016	
MirAncesTar	2016	
MiRNATIP	2016	
Mirza-G	2015	
Cupid	2015	
RNA22	2015	
mirbase	2014	
MirTar	2014	
MultiMiTar	2014	
MBStar	2014	
mirCoX	2013	
PACCMIT	2013	
BCmicrO	2012	
CoMeTa	2012	
DIANA	2012	
mirRcode	2012	
MirSNP	2012	
RepTar	*2011*	

microrna.org	*2008*	
ElMMo3	*2007*	
GenMir++	*2007*	
TargetRank	*2007*	
MAMI	*2006*	
PicTar	*2005*	
**Algorithm**	**Last update**	**Note**
RNAhybrid	2013	
MirMAP	2013	
miranda	2010	
BiTargeting	2010	
PITA	2008	
miTAR	*2021*	*failed to run*
TarPmiR	*2016*	*failed to run*
chimiRic	*2016*	*requires CLIP data*
miRNALasso	*2015*	*MATLAB based*
Avishkar	*2015*	*requires CLIP data*

*Excluded
